# Beyond willingness: unpacking pharmacists’ adoption of AI-driven clinical decision support systems through an extended UTAUT framework

**DOI:** 10.3389/fpubh.2026.1728867

**Published:** 2026-04-24

**Authors:** Yue Xie, Zhihui Song, Ente Wang, Ping Li, Jiawei Wang

**Affiliations:** Department of Pharmacy, Beijing Tongren Hospital, Capital Medical University, Beijing, China

**Keywords:** artificial intelligence–based clinical decision support system, pharmacist, structural equation modeling, technology acceptance, UTAUT

## Abstract

**Objective:**

Against the backdrop of the rapid integration of artificial intelligence–based clinical decision support systems (AI-CDSSs) into pharmacy practice, the actual acceptance of these systems by pharmacists and the underlying psychological and organizational mechanisms remain inadequately understood. Grounded in the unified theory of acceptance and use of technology (UTAUT), this study examines the factors influencing hospital pharmacists’ intention to use AI-CDSS and their subsequent usage behavior in China.

**Methods:**

The UTAUT framework was augmented by incorporating two additional constructs: technology trust and perceived risk. A cross-sectional survey was conducted using a structured questionnaire, and structural equation modeling (SEM) was employed to analyze the hypothesized relationships among variables.

**Results:**

The results indicated that performance expectancy (*β* = 0.308), effort expectancy (*β* = 0.274), and technology trust (*β* = 0.252) exerted significant positive effects on behavioral intention, whereas perceived risk showed a significant negative influence (*β* = −0.164). Social influence was not a significant predictor. Behavioral intention (*β* = 0.676) and facilitating conditions (*β* = 0.326) were both direct predictors of usage behavior.

**Conclusion:**

The acceptance of AI-CDSS among hospital pharmacists is primarily driven by perceptions of performance benefits, ease of use, and trust in the technology. These findings underscore the importance of enhancing the clinical value, algorithm transparency, and organizational support for AI-CDSS implementation. This study extends the theoretical applicability of UTAUT in high-stakes AI-mediated clinical environments.

## Introduction

### Background

With the rapid advancement of artificial intelligence (AI), its capabilities in analyzing and generating medical data have significantly improved, leading to widespread adoption in diverse clinical settings. AI-based clinical decision support systems (AI-CDSS) have been increasingly integrated into medical workflows and show considerable promise for overcoming the limitations of conventional treatment paradigms (1). In hospital pharmacy practice, AI-CDSS assists pharmacists in various critical tasks, including drug selection, dosage recommendation, identification of drug–drug interactions, and medication therapy management. This support is achieved by processing and interpreting multimodal patient data through machine learning algorithms and evidence-based medical knowledge. This integration enhances the accuracy of clinical judgment and reduces the risk of medication errors. Furthermore, AI-CDSS can facilitate medication verification, patient education, and automated generation of medical documentation, substantially streamlining workflow and increasing operational efficiency (2). Additionally, the system supports real-time monitoring of clinical parameters, enabling dynamic evaluation of therapeutic efficacy, early detection of prescription errors, and timely alerts for adverse drug events, thereby mitigating potential treatment risks (3).

In recent years, the Chinese government has demonstrated a consistent commitment to promoting the application and innovation of AI in healthcare. Particularly in pharmaceutical services, multiple policy documents have explicitly advocated the integration of AI technologies to advance rational drug use, optimize the allocation of medical resources, and improve patient experiences. Favorable policies and technological advances now lead experts to expect that implementing AI-CDSS will mitigate structural challenges (e.g., pharmacist shortages and uneven regional distribution) and will simultaneously drive drug-therapy management toward greater standardization, precision, and adoption (4).

Against this backdrop, understanding healthcare professionals’ acceptance of AI-CDSS has become a critical factor in facilitating their successful integration into clinical practice. The unified theory of acceptance and use of technology (UTAUT) offers a suitable theoretical framework for this type of research. First proposed by Venkatesh et al., UTAUT integrates key constructs from eight prominent technology adoption models, including the technology acceptance model (TAM) and the theory of reasoned action (TRA) (5). It delineates four core determinants, namely performance expectancy (PE), effort expectancy (EE), social influence (SI), and facilitating conditions (FC), along with moderating factors such as age, gender, and experience, to systematically explain an individual’s intention to use technology and subsequent usage behavior. Owing to its comprehensiveness and robust explanatory power, UTAUT has been widely applied in studies examining technology acceptance within healthcare settings (6).

The implementation of AI-CDSS involves technological sophistication, workflow transformation, and high-stakes clinical decision-making. Its adoption involves not only end-users’ perceptions and evaluations but may also be influenced by multi-level factors, such as organizational environment, resource availability, and regulatory requirements. Applying the UTAUT framework to analyze pharmacists’ attitudes and behavioral intentions toward AI-CDSS enables a multidimensional deconstruction of barriers and enablers in the adoption process. This approach provides a solid theoretical foundation for uncovering the underlying mechanisms shaping healthcare professionals’ acceptance of AI-CDSS (7).

### The role of performance expectancy variables in pharmacist adoption of AI-CDSS

Performance expectancy refers to the degree to which an individual believes that using a particular technology will enhance their job performance. In health care settings, this construct captures the extent to which physicians perceive that adopting an AI-CDSS can improve their clinical efficiency, diagnostic accuracy, or treatment outcomes. Studies identified PE as a critical predictor of healthcare personnel’s intention to use AI-CDSSs (8). This finding holds across various types of medical institutions, economic contexts, and professional groups.

However, the realization of PE often depends on the system’s ability to integrate seamlessly into clinical workflows. For instance, some health care professionals report that AI-CDSS may lack interoperability with existing electronic health records or impose disruptions in human-computer interaction, potentially counteracting efficiency gains (9). Moreover, the effectiveness of AI-CDSS in improving job performance is not yet universally validated, and perceptions of utility can vary based on specialty, setting, and system design (10, 11).

Furthermore, in addition to perceptions of efficiency, PE is critically determined by the technology’s capability to advance core clinical values, such as minimizing diagnostic errors, promoting patient safety, and enabling personalized treatment plans (12). When healthcare workers believe that an AI-CDSS can meaningfully augment their decision-making capabilities without undermining their autonomy, they are more likely to exhibit firm adoption intention (13).

### The role of effort expectancy variables in pharmacist adoption of AI-CDSS

Effort expectancy refers to an individual’s perception of the ease or difficulty associated with using a particular technology, including the anticipated effort and learning investment required. In healthcare settings, where complex workflows and time constraints are prevalent, users generally exhibit a strong preference for technologies that are intuitive, user-friendly, and seamlessly integrated into existing systems. Conversely, technologies that demand substantial time, effort, or involve a steep learning curve may negatively influence users’ acceptance and adoption behaviors (14).

Factors such as healthcare professionals’ prior familiarity with similar technologies and the intuitiveness of new system interfaces can significantly shape EE (15). Studies indicate that when AI-assisted decision-making systems are perceived as cumbersome to operate or poorly interoperable with hospital information systems, they may increase operational burden, particularly among pharmacists, and ultimately reduce willingness to use the technology (16).

### The role of social influence variables in pharmacist adoption of AI-CDSS

Social influence describes the expectations and pressures individuals perceive from significant others, such as colleagues, managers, or industry authorities, when deciding whether to adopt a new technology. This concept underscores the collective dimension of technology adoption behavior, highlighting how individuals shape their perceptions and adjust their behavioral intentions through social interactions. Within healthcare settings, SI typically operates through two primary pathways: first, via top-down transmission of institutional authority, such as mandatory hospital policies or administrative directives; and second, through horizontal influence based on professional consensus, including peer endorsement or recommendations from clinical experts. For instance, when pharmacists perceive strong institutional expectations to use an AI-CDSS, or when respected clinical experts publicly advocate for its use, considerable compliance pressure may arise (17).

Empirical studies have yielded mixed results regarding the impact of SI. Some evidence indicates that radiologists’ acceptance of AI-assisted imaging systems is significantly affected by SI (18). In contrast, other studies suggest that SI does not play a decisive role in healthcare professionals’ adoption of AI-CDSS (11). These inconsistencies may arise from differences in study populations, technology types, or organizational contexts, so the role of SI needs to be interpreted in the light of specific application settings.

### The role of facilitating conditions variables in pharmacist adoption of AI-CDSS

Facilitating conditions refer to the extent to which an individual perceives that organizations and infrastructures are available to support the use of a particular technology. These conditions reflect the external support and convenience provided for adopting technological systems. In the context of clinical decision support systems, facilitating conditions include a stable information technology infrastructure, data-integration capabilities, system interoperability, comprehensive training programs, and policy support. If a hospital lacks essential hardware resources, standardized data interfaces, or consistent technical maintenance, pharmacists who recognize the clinical value of the CDSS may still be less willing to use it because the implementation environment is poor. Moreover, low technology accessibility or insufficient team collaboration mechanisms can further adversely affect pharmacists’ acceptance of new technologies (19).

### The role of perceived risk variables in pharmacist adoption of AI-CDSS

Perceived risk denotes users’ anticipation and concerns regarding potential adverse outcomes associated with the adoption of new technologies. In the context of AI-CDSS, such risks encompass several dimensions, including diagnostic and treatment safety, patient privacy breaches, and ambiguities in legal liability (20). Risk perception varies among different groups of healthcare professionals. For instance, Lambert et al. reported that physicians express greater concern over patient privacy and data security, while nurses are more apprehensive about technology-related threats to treatment safety (21). These concerns undermine medical personnel’s willingness to adopt AI-CDSS by amplifying PR. Moreover, in cases where system errors occur, it becomes difficult to assign responsibility among users, developers, and providers of training data (22). As a result, healthcare professionals may face dual threats of litigation and professional reputational damage, which further exacerbates PR.

### The role of technical trust variables in pharmacist adoption of AI-CDSS

Technical trust refers to the confidence that users place in the reliability, accuracy, and overall technical competence of a technological system. In the context of AI-CDSS, TT encompasses three core dimensions: (a) System Transparency: This denotes the interpretability and traceability of AI-generated outputs, ensuring that the reasoning behind recommendations is accessible and verifiable by clinicians (23). (b) System Usability: This reflects the algorithmic robustness across diverse clinical scenarios, incorporating low data bias and seamless integration into existing workflows to support rather than disrupt clinical practice. (c) System Reliability: This encompasses the consistency of model performance, stability under varying conditions, and the sustained accuracy of predictions over time (24). Research indicates that AI-driven, dynamic algorithmic decision-making is increasingly supplanting traditional static rule-based systems. The reliability and stability of these AI-generated outputs have emerged as critical determinants influencing healthcare professionals’ acceptance and adoption of CDSS (10, 11)^.^

Researchers across multiple countries and medical specialties have empirically confirmed the explanatory power of UTAUT in predicting healthcare professionals’ adoption of AI technologies. However, research focusing on hospital pharmacists’ acceptance of AI-CDSS remains limited, and the factors influencing their willingness to adopt such technologies are not yet well understood. Therefore, this study applies the UTAUT framework to examine how PE, EE, SI, FC, TT, and PR collectively shape pharmacists’ adoption of AI-CDSS. Based on the UTAUT framework and the AI-CDSS context, we propose the following hypotheses:

H1: Performance expectancy (PE) positively influences behavioral intention (BI) to use AI-CDSS.

H2: Effort expectancy (EE) positively influences behavioral intention (BI) to use AI-CDSS.

H3: Social influence (SI) positively influences behavioral intention (BI) to use AI-CDSS.

H4 Perceived risk (PR) negatively influences behavioral intention (BI) to use AI-CDSS.

H5: Technical trust (TT) positively influences behavioral intention (BI) to use AI-CDSS.

H6: Behavioral intention (BI) positively influences use behavior (UB) of AI-CDSS.

H7: Facilitating conditions (FC) positively influence use behavior (UB) of AI-CDSS.

The study also aims to elucidate the potential pathways and strengths of these influences. By addressing these research gaps, this study aims to advance understanding of pharmacists’ acceptance of AI-CDSS and provide an evidence-based foundation for designing future interventions and related policies.

## Methods

### Method selection

This study employed a structured questionnaire to assess pharmacists’ acceptance of AI-CDSS. To ensure scale reliability and validity, a panel of four experts, comprising two clinical pharmacy experts, one health information technology specialist, and one statistical methodology expert, developed the questionnaire. It comprises two sections: the first collects sociodemographic and professional background information from participants, while the second incorporates the UTAUT framework. This section incorporates validated scales measuring four core constructs: PE, EE, SI, and FC. Additionally, to better align with the specific context of AI-CDSS, two extended variables, TT and PR, were included to provide a more comprehensive evaluation of pharmacists’ attitudes. Items in the second part were rated using a 5-point Likert scale, ranging from 1 (strongly disagree) to 5 (strongly agree). Before formal distribution, a pilot test was administered to 30 pharmacists; the questionnaire was subsequently revised based on their feedback and validity analysis. The final version consists of a sociodemographic profile and an AI-CDSS acceptance scale grounded in the UTAUT framework. The complete questionnaire is provided in Supplementary material. We adopted an anonymous questionnaire survey approach in this study, which minimized the psychological pressure on research participants to the greatest extent and thus facilitated the authentic reflection of pharmacists’ genuine barriers to adopting AI-CDSS.

### Data collection

From April 1 to August 30, 2025, 400 hospital pharmacists from 19 provinces in China were administered an electronic questionnaire on the Wenjuanxing platform on WeChat. To protect the quality of data put forth, each WeChat account was allowed a single questionnaire submission to prevent duplicate responses. No personally identifiable information was collected, and IP addresses were not tracked. Consequently, it was not possible to identify individual survey responses or link responses to specific participants. Responses identified to have insufficient effort responding were excluded from analysis, namely those responses that had been completed in fewer than 120 s. Minimum time thresholds were derived based upon aggregating the required minimum reading durations of background information (2 s) and AI usage intention (4.5 s) from the questionnaire. The intent of the design is to provide all respondents with adequate time to read and begin understanding all questionnaire items (25).

After collection, the data were downloaded and collated in a single standard format. All latent variables were measured on a 5-point Likert scale. Dimensional scores were computed by averaging their corresponding items, with higher scores representing a higher level of the respective construct. We performed all data processing and statistical analyses with SPSS 26.0 and R 4.5.1. SPSS supplied the descriptive statistics and the reliability and validity tests; R provided the confirmatory factor analysis, structural equation modelling, and path coefficient estimates. Factor loadings were derived from Confirmatory Factor Analysis using Maximum Likelihood estimation with robust standard errors in SPSS 26.0. Standardized factor loadings represent the correlation between each observed item and its underlying latent construct, with values ≥0.70 considered satisfactory (26). Statistical significance for the hypothesis tests was defined using a benchmark of *p* < 0.05.

### Ethical considerations

This study was conducted in accordance with the principles of the Declaration of Helsinki and received approval from the Capital Medical University of Beijing Tongren Hospital Clinical Research Ethics Committee (Approval No. TREC2025-KY174). We obtained written informed consent from every participant before enrollment and handled all data confidentially; no personally identifiable information was disclosed.

## Results

**Figure 1:**
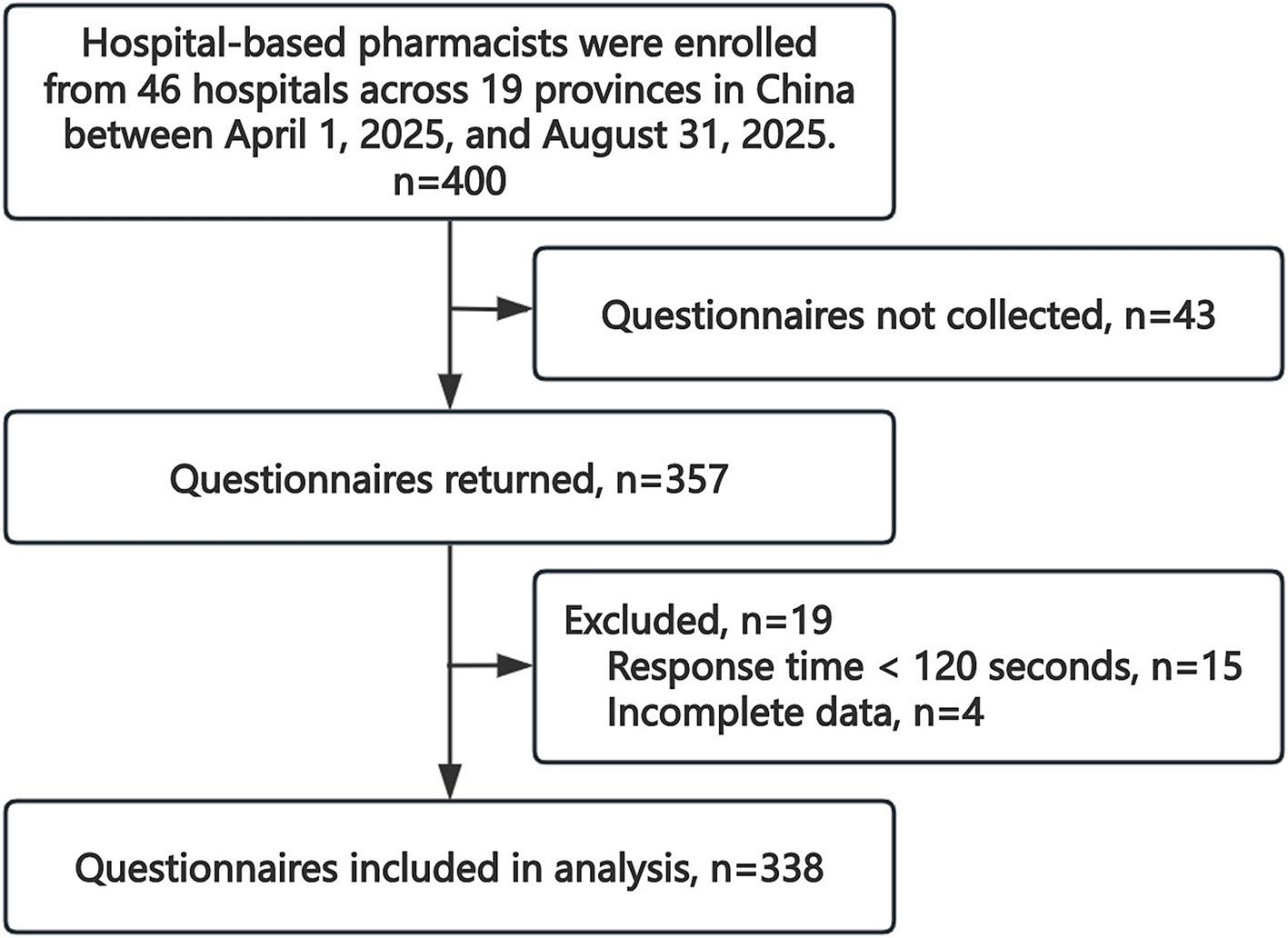
Flowchart of the questionnaire screening process.

We distributed 400 questionnaires and received 357 responses. After review, 19 questionnaires were excluded due to incomplete responses, yielding 338 valid questionnaires and an effective response rate of 79.33%. Figure 1 summarizes the screening process for questionnaire inclusion. Table 1 summarizes the demographic characteristics of the participating pharmacists. The majority of respondents were female. In terms of age distribution, most participants (69.23%) were between 25 and 45 years old. Among the respondents, 41.12% held intermediate professional titles and 59.76% worked in tertiary medical institutions, 24.26% were from North China, and 22.78% were from East China. In the survey on AI-CDSS usage frequency, the largest proportion of pharmacists reported using it daily, accounting for 31.07%.

**Table 1:** Demographic characteristics of the sample.

Category	*N* (%)
Gender	
Male	86 (25.44%)
Female	252 (74.56%)
Age (years)	
≤25	5 (1.48%)
>25–35	106 (31.36%)
>35–45	128 (37.87%)
>45–55	77 (22.78%)
>55	22 (6.51%)
Major title	
Clinical pharmacist I	97 (28.70%)
Clinical pharmacist II	139 (41.12%)
Clinical pharmacy specialist	75 (22.19%)
Senior clinical pharmacy	27 (7.99%)
Hospital grade	
Primary	55 (16.27%)
Secondary	81 (23.96%)
Tertiary	202 (59.76%)
Geographical distribution	
Northeast China	54 (15.98%)
North China	82 (24.26%)
East China	67 (22.78%)
Central and Southern China	60 (17.75%)
Southwest China	29 (8.58%)
Northwest China	46 (10.65%)
AI-CDSS usage experience	
Never used	16 (4.73%)
≤3 times per month	60 (17.75%)
1–2 times per week	69 (20.41%)
≥3 times per week	88 (26.04%)
Daily	105 (31.07%)

AI-CDSS, AI-based clinical decision support systems.

We evaluated the scale’s reliability and validity using Cronbach’s *α*, composite reliability (CR), and average variance extracted (AVE). As presented in Table 2, the Cronbach’s α values for the eight latent variables ranged from 0.744 to 0.933, and the CR values ranged from 0.781 to 0.957. Both measures exceeded the conventional threshold of 0.7, indicating satisfactory internal consistency. Furthermore, the AVE values fell between 0.621 and 0.861, all above the recommended criterion of 0.5, which supports adequate convergent validity for each latent variable (27).

**Table 2:** Reliability and validity of the questionnaire.

Variable and items	Factor loading	Cronbach α	CR	AVE
Performance expectancy		0.933	0.957	0.861
PE1	0.838			
PE2	0.901			
PE3	0.855			
Effort expectancy		0.792	0.925	0.783
EE1	0.728			
EE2	0.658			
EE3	0.702			
Social influences		0.862	0.890	0.759
SI1	0.739			
SI2	0.750			
SI3	0.713			
Facilitating conditions		0.859	0.892	0.736
FC1	0.758			
FC2	0.773			
FC3	0.667			
Perceived risk		0.849	0.910	0.786
PR1	0.803			
PR2	0.837			
PR3	0.742			
Technology trust		0.772	0.868	0.717
TT1	0.628			
TT2	0.671			
TT3	0.703			
Behavioral intention		0.883	0.931	0.804
BI1	0.804			
BI2	0.758			
BI3	0.835			
Usage behavior		0.744	0.781	0.621
UB1	0.704			
UB2	0.633			
UB3	0.676			

AVE, average variance extracted; BI, behavioral intention; CR, composite reliability; EE, effort expectancy; FC, facilitating conditions; PE, performance expectancy; PR, perceived risk; SI, social influences; TT, technology trust; UB, usage behavior.

PE1–PE3, performance expectancy items 1–3; EE1–EE3, effort expectancy items 1–3; SI1–SI3, social influence items 1–3; FC1–FC3, facilitating conditions items 1–3; BI1–BI3, behavioral intention items 1–3; UB1–UB3, use behavior items 1–3. Full item wording is provided in Supplementary material.

Table 3 summarizes the assessment of discriminant validity. The diagonal entries represent the square roots of the average variance extracted (AVE) for each latent variable, while the off-diagonal elements denote the correlation coefficients between constructs. Results show that the square root of the AVE for each construct ranged from 0.621 to 0.861, exceeding the absolute correlation coefficients with other constructs, thus satisfying the Fornell-Larcker criterion and supporting discriminant validity (27).

**Table 3:** Assessment of discriminant validity.

Variable	PE	EE	SI	FC	PR	TT	BI	UB
PE	0.861							
EE	0.559**	0.783						
SI	0.630**	0.528**	0.759					
FC	0.546**	0.676**	0.717**	0.736				
PR	−0.453*	−0.529**	−0.573*	−0.532**	0.786			
TT	0.645**	0.598**	0.529**	0.682**	−0.581*	0.717		
BI	0.722	0.645 **	0.539*	0.423*	−0.482**	0.694**	0.804	
UB	0.549	0.561	0.602	0.556	−0.497	0.585	0.614*	0.621

BI, behavioral intention; EE, effort expectancy; FC, facilitating conditions; PE, performance expectancy; PR, perceived risk; SI, social influences; TT, technology trust; UB, usage behavior.

**Correlation is significant at the 0.01 level (2-tailed); *Correlation is significant at the 0.01 level (2-tailed).

Correlation analysis revealed positive correlations among PE, EE, SI, and FC, with coefficients ranging from 0.423 to 0.717. Each of these constructs was also positively associated with behavioral intention (BI) and usage behavior (UB). In contrast, PR demonstrated negative correlations with all other core constructs (*r* = −0.453 to −0.581). BI and UB showed a significant positive correlation (*r* = 0.614, *p* < 0.05). Overall, these findings provide evidence of adequate discriminant validity and theoretical consistency at the construct level in the research model.

**Figure 2:**
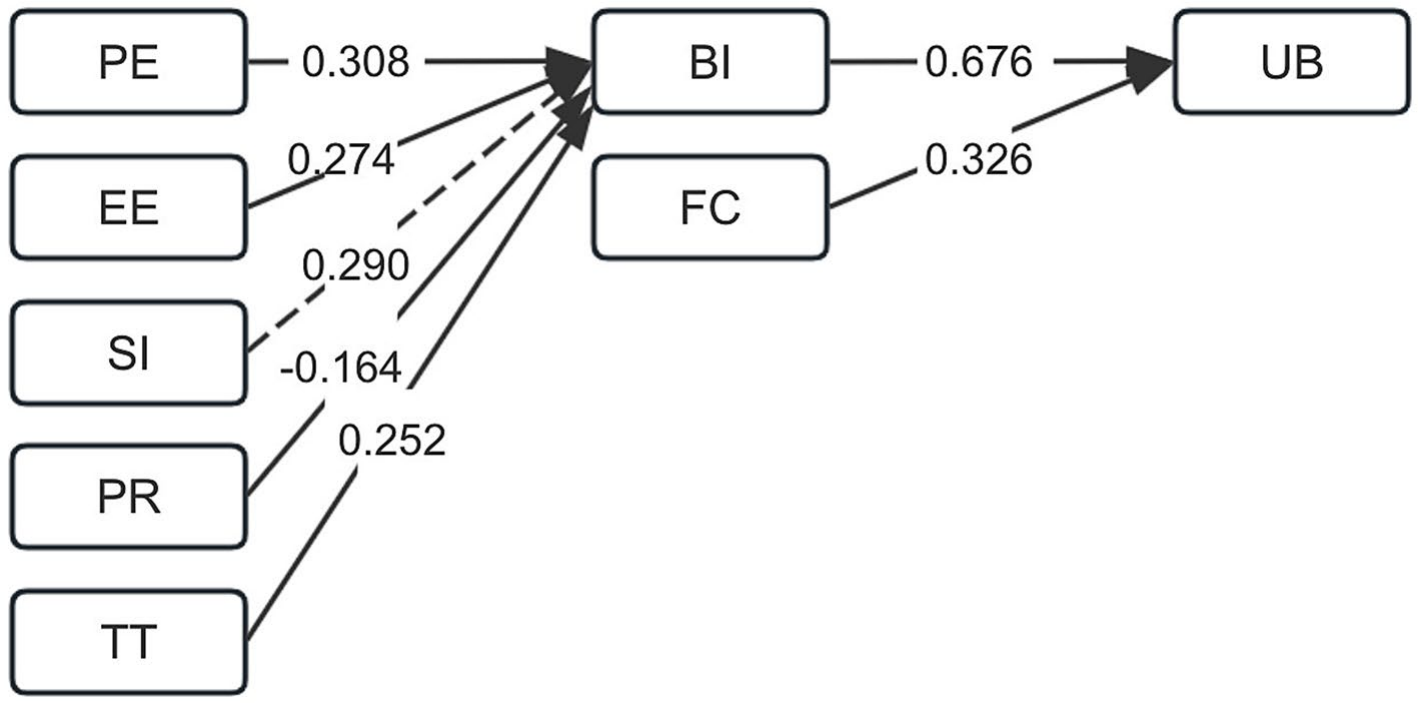
Path coefficients for the final structural equation model. Abbreviation BI, behavioral intention; EE, effort expectancy; FC, facilitating conditions; PE, performance expectancy; PR, perceived risk; SI, social influences; TT, technology trust; UB, usage behavior.

This study employed structural equation modeling to examine the proposed theoretical hypotheses (H1–H7). The model demonstrated a good fit to the data, with the following indices: chi-square/degrees of freedom (χ^2^/df) = 2.380, *p* = 0.086, root mean square error of approximation (RMSEA) = 0.069 [90% confidence interval (CI): 0.053–0.087], comparative fit index (CFI) = 0.955, Tucker-Lewis index (TLI) = 0.923, and standardized root mean square residual (SRMR) = 0.067. As summarized in Table 4 and Figure 2, the standardized path coefficients revealed H1 (PE → BI), H2 (EE → BI), and H5 (TT → BI) were supported, with significant positive effects (*p* < 0.01). In contrast, H4 (PR → BI) was supported but showed a significant negative effect (*p* < 0.01), as hypothesized. However, H3 (SI → BI) failed to reach significance (*p* = 0.053) and was therefore not supported. In addition, both H6 (BI → UB) and H7 (FC → UB) were confirmed as significant direct predictors (*p* < 0.01).

**Table 4:** Standardized path coefficients for the final model.

Relationship	Estimate	*P*	SE	CR	Comment	Result
BI→UB	0.676	<0.01	0.195	3.460	Significant	Supported
FC→UB	0.326	<0.01	0.118	2.759	Significant	Supported
PE→BI	0.308	<0.01	0.094	3.283	Significant	Supported
EE→BI	0.274	<0.01	0.075	3.652	Significant	Supported
SI→BI	0.290	0.053	0.191	1.519	Not significant	Not supported
PR→BI	−0.164	<0.01	0.073	−2.250	Significant	Supported
TT→BI	0.252	<0.01	0.062	4.054	Significant	Supported

BI, behavioral intention; CR, critical ratio; EE, effort expectancy; FC, facilitating conditions; PE, performance expectancy; PR, perceived risk; SE, standard error; SI, social influences; TT, technology trust; UB, usage behavior.

that PE, EE, and TT exerted significant positive effects on BI (*p* < 0.01). In contrast, PR had a significant adverse impact (*p* < 0.01). These results support the corresponding hypotheses. However, SI failed to reach significance as a predictor of BI (*p* = 0.053), so the corresponding hypothesis was not supported. In addition, both BI and FC were significant direct predictors of UB (*p* < 0.01), confirming the proposed relationships.

## Discussion

To the best of our knowledge, this study represents the first application of the UTAUT to investigate hospital pharmacists’ acceptance of AI-CDSS, thereby extending the applicability of this theoretical framework to these specialized healthcare professionals. Using survey data collected from 338 pharmacists, we incorporated two additional constructs, technology trust and PR, into the original UTAUT model and evaluated the hypothesized relationships through structural equation modeling (SEM). The findings reveal that PE, EE, and TT significantly positively influence pharmacists’ BI to use AI-CDSS, whereas PR exerts a significant adverse effect. BI and FC both exerted a significant direct effect on actual use behavior. Furthermore, SI demonstrated a relatively weak impact on usage intention within this highly specialized professional cohort.

### Joint influence of performance and effort expectancy

This study demonstrates that both PE and EE significantly predict pharmacists’ intention to use AI-CDSS, aligning with established technology acceptance theories. Despite the technological novelty and inherent complexity of AI-CDSS, users’ adoption behaviors remain strongly influenced by their perception of the system’s practical utility (28, 29).

As essential participants in evidence-based practice, pharmacists are primarily responsible for ensuring prescription accuracy, medication safety, and treatment efficacy (30). AI-CDSS supports these core duties by offering intelligent prescription review, individualized dosing recommendations, and adverse drug reaction alerts. These functions directly enhance review quality, reduce medication errors, and optimize therapeutic outcomes. Consequently, this strengthens pharmacists’ professional identity and role satisfaction. Thus, the belief that AI-CDSS substantially improves job performance constitutes a fundamental motivator for its acceptance among pharmacists.

Similarly, EE is expected to exert a significant positive influence on the intention to use AI-CDSS. This implies that systems designed to be user-friendly, intuitive, and quick to master are more likely to be adopted by pharmacists. Given that pharmacists often work in high-intensity, fast-paced environments, the time available for learning and adapting to new systems is typically limited (31). An AI-CDSS that has a complex interface, responds slowly, or integrates poorly with hospital information systems such as HIS or EMR is likely to be viewed as an added burden, regardless of the quality of its clinical decision support. Such perceptions could reduce EE and ultimately diminish pharmacists’ intention to use the system. Conversely, systems with streamlined interfaces, clear workflows, and low cognitive demands can elevate EE, thereby fostering acceptance. These observations align with prior studies on technology acceptance among healthcare professionals and reinforce the role of EE as a key psychological mechanism that facilitates technology adoption (10).

### The facilitative role of technology trust

The findings of this present study demonstrate that technology trust exerts a significant positive influence on pharmacists’ willingness to use AI-CDSS. This result aligns with prior research on AI technology acceptance within the global healthcare sector. In clinical applications of AI-CDSS, which directly impact patient safety and health outcomes, pharmacists’ trust in the system’s algorithmic reliability, fairness, and logic outweighs conventional TT factors typically emphasized in traditional technology acceptance models. Instead, it emerges as a critical determinant shaping adoption intentions. This insight enhances the understanding of technology acceptance mechanisms in high-risk and high-uncertainty clinical environments (32).

Consistent with our observations, Choung et al. indicated that technology trust fosters healthcare professionals’ intention to use AI systems by mitigating cognitive uncertainty (33). In pharmacy practice, where pharmacists serve as guardians of medication safety, their trust in AI-CDSS is primarily built upon the system’s accuracy and clinical relevance in areas such as drug selection, dosage optimization, drug interaction alerts, and error prevention. Additionally, aligning system recommendations with evidence-based guidelines and professional medical literature substantially contributes to trust formation. Nevertheless, the ‘black box’ nature of many AI algorithms, which often limits interpretability, remains a barrier to building robust trust in technology. To address this challenge, future development of AI-CDSS should emphasize enhancing algorithmic interpretability through approaches such as visualizing decision pathways, providing supporting evidence, or displaying confidence scores. Such measures are likely to strengthen pharmacists’ trust and facilitate the integration of AI-driven tools into clinical workflows.

### The inhibiting role of perceived risk

This study demonstrates that perceived risk exerts a significant negative influence on pharmacists’ willingness to use AI-CDS. The PR manifests primarily in three dimensions: data privacy, algorithmic fairness, and medical liability attribution (30). Given the highly sensitive nature of medical data, pharmacists are particularly concerned about data security and privacy protection. AI systems handling such data must adhere strictly to legal and regulatory standards to ensure compliance and prevent breaches, thereby alleviating pharmacists’ apprehensions.

In terms of algorithmic fairness, potential biases may arise if training datasets are unrepresentative across key demographic variables such as race, age, or gender, which could introduce systematic errors into AI-driven decisions and exacerbate healthcare disparities. Moreover, the deployment of AI-CDSS in clinical settings raises unresolved questions regarding accountability in cases of medical errors. The ambiguity surrounding legal liability significantly heightens pharmacists’ PR. Previous surveys indicate that 48.8% of healthcare professionals express concerns regarding the ethical safety and algorithmic fairness of AI technologies, while 80% believe that these aspects require further improvement (34). Therefore, strengthening data privacy safeguards, enhancing algorithmic fairness, and clarifying liability allocation rules are critical to mitigating PR and improving pharmacists’ acceptance of AI-CDSS.

### Heterogeneity of social impact

The results of this study show that social influence has a standardized effect on behavioral intention (*β* = 0.290) comparable to that of EE (*β* = 0.274) and TT (*β* = 0.252), but it is demonstrated a marginally non-significant (*p* = 0.053, SE = 0.191). This suggests that social influence may affect pharmacists’ willingness to use AI-CDSS, but there is considerable inter-individual heterogeneity in this sample (35).

This might be attributed to the fact that AI-CDSS in hospital pharmacy is still an emerging technology without relevant institution-use standards or professional general agreement fully established. However, pharmacists are professionals in medication safety who have received consistent training and are ultimately accountable for medication safety, and they use relevant technical-use guidelines as an important basis for their clinical practice (36). Currently, the absence of clear standards means pharmacists lack a consensus reference for behavior. Consequently, individual perceptions of ‘peer expectations’ and ‘organizational requirements’ vary substantially, which amplifies variation in the specific SI effect.

Accordingly, healthcare managers should not overlook the potential role of social influence based solely on the marginal effects of statistical significance. It is recommended that healthcare managers promote the formation of normative consensus by establishing standards for AI-CDSS use, setting up peer review mechanisms, and promoting successful cases. We expect that as technology diffusion matures and institutional policies become clearer, the relationship between SI and BI will become further clarified in future research.

### The dual influence on use behavior

This study demonstrated a significant positive effect of behavioral intention on actual use behavior (path coefficient *β* = 0.676, *p* < 0.001), aligning with the core proposition of the Unified Theory of Acceptance and Use of Technology (UTAUT). BI reflecting an individual’s readiness to adopt a technology, serves as a direct and robust predictor of actual behavior.

Additionally, FC exerted a substantial direct influence on UB (*β* = 0.326, *p* < 0.01), consistent with the findings reported by English et al. in their study on clinical pharmacists’ adoption of Clinical Decision Support Systems (CDSS) (37). FC encompasses organizational support mechanisms such as hardware infrastructure, technical assistance, training programs, and seamless integration with existing workflows. The results show that high behavioral intention alone does not guarantee sustained use; inadequate hardware, software, or timely technical support can still prevent intention from translating into stable actual use. Thus, FC act as a critical bridge between “willingness to use” and “actual use,” even extending to “continued use,” and serve as an essential organizational guarantee for the successful implementation and scaling of AI-CDSS. FC exerted a significant direct effect on UB even after controlling for BI, indicating that adequate infrastructure, technical support and streamlined workflows can offset shortfalls in individual motivation. We therefore recommend a two-pronged approach: enhance intention by improving system reliability, usability and perceived value, and concurrently upgrade infrastructure and support services to create an environment that sustains long-term adoption of the AI-CDSS.

The study sample encompassed all six administrative geographical regions of China, with a distribution profile consistent with the macro-level geographic distribution of pharmacists reported by Qiao et al. (38). This demonstrates that our sample did not suffer from a significant geographical concentration bias and captured a reasonably diverse range of practice environments across the country.

Notably, Our sample comprised a higher proportion of pharmacists from tertiary hospitals. Although this distribution differs from the national profile of healthcare institutions, it should be interpreted within the context of technology diffusion. Currently, artificial intelligence clinical decision support systems (AI-CDSS) are in the early stages of diffusion, having been piloted and applied in select hospitals but not yet scaled nationwide (39). Consequently, our sample’s composition provides a valuable and focused snapshot of the ‘early adopter’ and ‘early majority’ pharmacist populations. This group are most likely to be currently interacting with or anticipating the arrival of AI-CDSS. This sampling strategy mitigates the risk of underestimating genuine adoption barriers that could result from including respondents unfamiliar with the technology (40). Thus, our study aims to provide actionable insights for current technology implementers rather than seeking early generalization to contexts remain inadequately prepared.

### Heterogeneity associated with individual background characteristics

In this study, individual factors such as age, educational background, professional experience, and baseline AI understanding may influence pharmacists’ willingness to adopt AI-CDSS (41–43). Younger or less experienced pharmacists may perceive AI-CDSS as a tool to compensate for their limited experience, thereby demonstrating higher initial adoption intentions. Those with innovative research backgrounds or prior AI exposure may possess stronger analytical competencies, enabling more critical evaluation of AI-generated recommendations and fostering more cautious trust. Older pharmacists with extensive clinical experience but limited AI exposure may weigh AI recommendations against long-accumulated clinical wisdom. Therefore, the willingness to adopt AI-CDSS may be heterogeneous among pharmacists with different backgrounds. Future research should incorporate comprehensive measures of age, educational background, professional experience, and baseline AI literacy to examine whether UTAUT constructs in pharmacists’ acceptance of AI-CDSS are moderated by individual background characteristics, thereby providing practical guidance for deploying AI-CDSS in diverse pharmacy settings with varied demographic and professional profiles.

### Limitations

Firstly, due to limitations in resources and time, the proportion of tertiary hospitals in this study sample was high. These hospitals usually have more advanced information technology infrastructure, which may limit the generalizability of the study results to pharmacists in non-tertiary hospitals. Second, although this research extended an existing theoretical framework by incorporating the variables of “technology trust” and “perceived risk,” the selection of these constructs was primarily literature-driven. Other potentially relevant variables, such as personal innovativeness or technology anxiety, were not considered. Future research could purposefully recruit samples from different grade of healthcare institutions to assess the situation of specific constructs in the UTAUT model across different levels of healthcare institutions. Additionally, employing qualitative approaches like in-depth interviews would help uncover underlying factors more systematically.

## Conclusion

This study validates the applicability of the UTAUT model in explaining pharmacists’ BI to adopt AI-CDSS. The results demonstrate that PE, EE, and TT exert significant positive effects on pharmacists’ adoption intention, whereas PR serves as a critical barrier. Furthermore, FC and BI directly influence actual UB. Therefore, we propose several evidence-based strategies to facilitate the seamless integration of AI-CDSS into routine pharmacy workflows. First, highlighting clinical value, such as through precision medication recommendations and adverse drug reaction alerts, can enhance performance expectancy. Second, interfaces must be designed for clarity and simplicity so that users can operate the system with minimal effort. Third, improving algorithmic transparency and interpretability will help strengthen technology trust. Furthermore, it is essential to enhance data security measures and ensure strict ethical compliance. Clearly delineating responsibilities for potential risks will thereby reduce users’ PR. Future studies may adopt multicenter cohort designs and incorporate additional psychological constructs, such as technology anxiety and personal innovativeness, to further elucidate factors influencing the targeted implementation of AI-CDSS.

## Data Availability

The raw data supporting the conclusions of this article will be made available by the authors, without undue reservation.
