# Supplementary material for: Beyond willingness: unpacking pharmacists’ adoption of AI-driven clinical decision support systems through an extended UTAUT framework
Source: Front Public Health. 2026 Apr 24;14:1728867. doi: 10.3389/fpubh.2026.1728867 (PMC13152858; doi:10.3389/fpubh.2026.1728867)
Supplement: Supplementary file 1 [file Data_Sheet_1.PDF]

Supplementary

Survey on Pharmacists' Adoption of AI-Driven Clinical Decision Support Systems

Part 1: Demographic and Professional Background
Instructions: Please provide the following information about yourself.

1.Gender
○ Male
○ Female

2.Age
○ ≤ 25 years old
○ ＞ 25-35 years old
○ ＞ 35-45 years old
○ ＞ 45-55 years old
○ ＞ 55 years old

3. Your major title is
○ Clinical Pharmacist I
○ Clinical Pharmacist II
○ Clinical Pharmacy Specialist
○ Senior Clinical Pharmacy

4.Name of your healthcare institution: __________

5. Grade of your healthcare institution
○ Primary
○ Secondary
○ Tertiary

6. The province where your healthcare institution is located __________

7. Your frequency of using artificial intelligence–based clinical decision support systems (AI-CDSS) is
○ Never used
○ ≤ 3 times per month
○ 1–2 times per week
○ ≥ 3 times per week
○ Daily

Part 2: Familiarity and Experience with AI Technology
Instructions: Please indicate your level of agreement with the following statements by checking the response that best reflects your opinion. Use the scale below as your guide：1 = strongly disagree, 2 = disagree, 3 = neutral, 4 = agree, 5 = strongly agree

Factors	Variables	Items	1	2	3	4	5
Performance Expectancy	PE1	8. I believe AI-CDSS can improve my work efficiency in prescription/medication order review.	○	○	○	○	○
	PE2	9. AI-CDSS can help me identify medication safety issues more quickly, such as medication errors, adverse drug reactions, or drug-drug interactions.	○	○	○	○	○

	PE3	10. AI-CDSS is helpful for me to better evaluate drug efficacy issues, such as symptom relief, improvements in laboratory tests, or achievement of treatment goals.	○	○	○	○	○
Effort Expectancy	EE1	11. Learning to operate the AI-CDSS is easy for me.	○	○	○	○	○
	EE2	12. The system interface of the AI tools I have used is clear and intuitive, facilitating my understanding and operation.	○	○	○	○	○
	EE3	13. I believe AI-CDSS can be seamlessly integrated into existing workflows; it is simple to operate and runs smoothly.	○	○	○	○	○
Social Influences	SI1	14. My colleagues generally believe that pharmacists should actively adopt AI-CDSS in their practice.	○	○	○	○	○
	SI2	15. The management of my hospital actively supports the implementation of AI-CDSS in pharmaceutical services.	○	○	○	○	○
	SI3	16. Patients in my practice setting demonstrate high acceptance of AI-assisted pharmaceutical services.	○	○	○	○	○
Facilitating Conditions	FC1	17. My hospital provides sufficient training resources for the use of AI-CDSS.	○	○	○	○	○
	FC2	18. I can obtain timely technical support when encountering problems with the AI-CDSS.	○	○	○	○	○
	FC3	19. The existing hardware infrastructure (e.g., computers, network servers) in my institution is adequate to support the operation of AI technologies.	○	○	○	○	○
Perceived Risk	PR1	20. I am concerned that AI-CDSS technology may lead to medication safety issues.	○	○	○	○	○
	PR2	21. I am concerned that AI-CDSS may compromise patient data privacy.	○	○	○	○	○
	PR3	22. I am concerned that over-reliance on AI-CDSS could result in the deterioration of my professional competence.	○	○	○	○	○
Technology Trust	TT1	23. I trust that AI-CDSS outputs are reliable and adhere to clinical technical specifications.	○	○	○	○	○
	TT2	24. I trust that AI-CDSS outputs are operable and aligned with clinical practice requirements.	○	○	○	○	○
	TT3	25. I trust that AI-CDSS outputs are consistent and reproducible.	○	○	○	○	○
Behavioral Intention	BI1	26. I intend to use the AI-CDSS regularly in my clinical practice.	○	○	○	○	○
	BI2	27. I plan to refer the AI-CDSS recommendations in my work.	○	○	○	○	○
	BI3	28. I would recommend the AI-CDSS to my clinical colleagues.	○	○	○	○	○
Usage Behavior	UB1	29. I view myself as a frequent user of AI-CDSS.	○	○	○	○	○
	UB2	20. I am inclined to use AI-CDSS when given the opportunity.	○	○	○	○	○
	UB3	31. I tend to utilize AI-CDSS for the majority of my clinical tasks.	○	○	○	○	○

Abbreviation： BI = behavioral intention, EE = effort expectancy, FC = facilitating conditions, PE = performance expectancy, PR = perceived risk, SI = social influences, TT = technology trust, UB = usage behavior.
